# Leaky Gut as a Potential Culprit for the Paradoxical Dysglycemic Response to Gastric Bypass-Associated Ileal Microbiota

**DOI:** 10.3390/metabo11030153

**Published:** 2021-03-08

**Authors:** Mohammed K. Hankir, Florian Seyfried, Isabel N. Schellinger, Nicolas Schlegel, Tulika Arora

**Affiliations:** 1 Department of General, Visceral, Transplant, Vascular and Pediatric Surgery, University Hospital Würzburg, Oberdürrbacherstraße 6, 97080 Würzburg, Germany; seyfried_f@ukw.de (F.S.); schlegel_n@ukw.de (N.S.); 2 Department of Endocrinology and Nephrology, University Hospital Leipzig, Liebigstraße 20, 04103 Leipzig, Germany; isabel@schellinger-research.de; 3 Novo Nordisk Foundation Center for Basic Metabolic Research, University of Copenhagen, Blegdamsvej 3B, 2200 København, Denmark

**Keywords:** Roux-en-Y gastric bypass surgery, intestinal microbiota, intestinal epithelial barrier, systemic endotoxemia, type 2 diabetes

## Abstract

Altered host-intestinal microbiota interactions are increasingly implicated in the metabolic benefits of Roux-en-Y gastric bypass (RYGB) surgery. We previously found, however, that RYGB-associated ileal microbiota can paradoxically impair host glycemic control when transferred to germ-free mice. Here we present complementary evidence suggesting that this could be due to the heightened development of systemic endotoxemia. Consistently, application of ileal content from RYGB-treated compared with sham-operated rats onto Caco-2 cell monolayers compromised barrier function and decreased expression of the barrier-stabilizing proteins claudin-4 and desmoglein-2. Our findings raise the possibility that RYGB-associated ileal microbiota produce and release soluble metabolites which locally increase intestinal permeability to promote systemic endotoxemia-induced insulin resistance, with potential implications for the treatment of RYGB patients who eventually relapse onto type 2 diabetes.

## 1. Introduction

Roux-en-Y gastric bypass (RYGB) is presently one of the most frequently performed and effective bariatric surgical procedures, and causes long-term remission of type 2 diabetes in up to 40% of cases [1]. By reducing food intake and redirecting its flow from the stomach directly to the mid-jejunum, as well as redirecting the flow of digestive secretions such as undiluted bile and gastric acid yet further down the small intestine, RYGB has a profound impact on the composition and diversity of microbial communities residing throughout the length of the gastrointestinal tract [2,3]. This has been proposed to play a decisive role in improving glycemic control post-operatively through the action of various microbial metabolites such as secondary bile acids [4], and has received perhaps the strongest support from intestinal microbiota transfer experiments performed on germ-free mice [2,3,5]. For example, we previously showed that transfer of the cecal microbiota from RYGB-treated compared with sham-operated Zucker Fatty *fa/fa* rats, which are severely obese and glucose intolerant due to a loss-of-function mutation in the leptin receptor gene [6], to germ-free mice conferred them with lower peak blood glucose levels during an oral glucose tolerance test (OGTT) [2]. Paradoxically however, transfer of the ileal microbiota from the same donors to germ-free mice exerted the opposite effect, and markedly impaired oral glucose tolerance [2]. While the recipients of ileal microbiota from RYGB-treated compared with sham-operated rats had greater adiposity [2], which could explain a development of insulin resistance, the underlying molecular and cellular bases for their dysglycemia were at the time unclear. 

The translocation of pro-inflammatory, gram-negative bacterial species and/or their products such as lipopolysaccaride (LPS) from the intestinal lumen to the general circulation is known as systemic endotoxemia, and is an increasingly recognized etiological factor of obesity-associated insulin resistance and type 2 diabetes [7]. Systemic endotoxemia is thought to arise, in part, from unfavorable changes in the intestinal microbiota from prolonged consumption of a high-fat diet and their reduced production of intestinal epithelial barrier (IEB)-stabilizing metabolites [8]. Compounding matters further, this unfavorable change in the intestinal microbiota also elicits a highly orchestrated local immune cell response involving the release pro-inflammatory, IEB-disruptive cytokines [9]. Various interventions that stabilize the IEB to attenuate systemic endotoxemia in obesity, including small molecule drugs [8,9] and *Akkermansia muciniphila* transplant [8,10], are thus being considered for treating the associated insulin resistance and type 2 diabetes. Clinical [11,12,13] and preclinical [14,15] studies have further shown that RYGB attenuates systemic endotoxemia along with the improvements in glycemic control. We therefore turned our attention to the IEB as a potential target of RYGB-associated ileal microbiota. 

## 2. Results

### 2.1. Ileal Content Transfer from RYGB-Treated Rats to Germ-Free Mice Modestly Impairs Oral Glucose Tolerance and Heightens Systemic Endotoxemia Development

**Figure 1:**
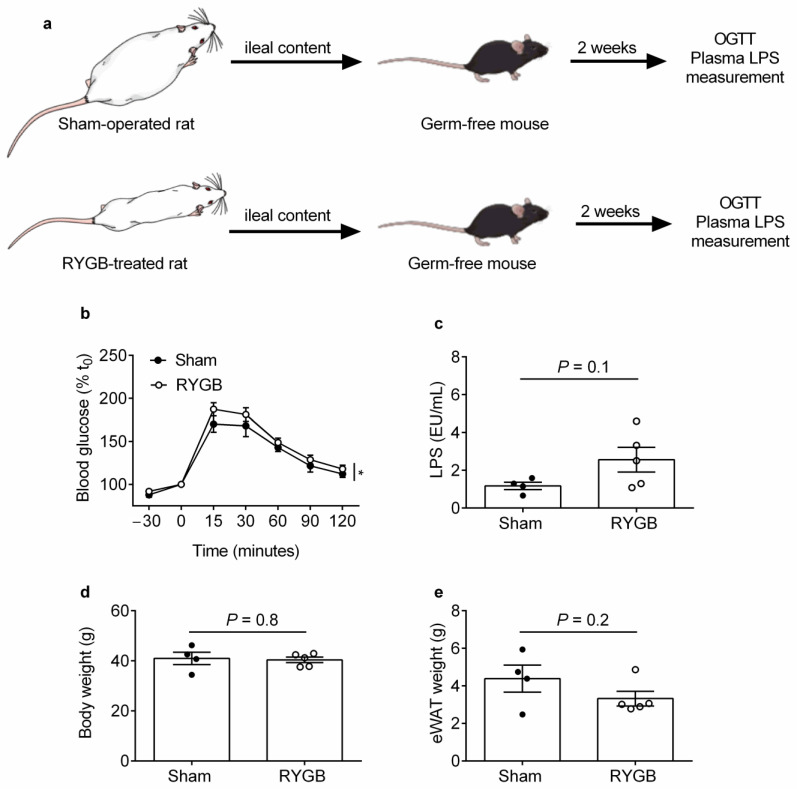
Ileal Content Transfer from RYGB-Treated Rats to Germ-Free Mice Modestly Impairs Oral Glucose Tolerance and Heightens Systemic Endotoxemia Development (**a**) Schematic diagram illustrating the ileal content transfer experiment performed on germ-free mice. (**b**) Percentage (%) change in tail vein blood glucose concentrations relative to *t* = 0 min (t_0_) during the oral glucose tolerance test of germ-free mice that had received ileal content from RYGB-treated and sham-operated rats. (**c**) Plasma LPS concentrations, (**d**) body weight and (**e**) epididymal white adipose tissue (eWAT) weight from the mice in (**b**). *n* = 4–5 mice/group. Data are presented as mean ± SEM. Statistical significance was determined by two-way ANOVA (main effect of treatment) in (**b**) and two-tailed, unpaired *t*-test in (**c**–**e**). * *p* < 0.05.

We first wanted to replicate our previous findings [2] and show that ileal content transfer from a separate cohort of RYGB-treated compared with sham-operated rats to germ-free mice impairs oral glucose tolerance. Ileal content was obtained 6 weeks after surgeries (body weight change from baseline of −12.1 ± 2.3% vs. +22.8 ± 3.2%, and average daily energy intake of 73.2 ± 5.1 kcal vs. 104.9 ± 2.9 kcal, for RYGB-treated vs. sham-operated rats, respectively, *p* < 0.0001 for both comparisons) and was orally gavaged into germ-free mice as previously described [2] (Figure 1a). 

In contrast with our previous findings [2], germ-free mice that received ileal content from RYGB-treated compared with sham-operated rats had similar blood glucose levels during the OGTT (Figure S1). This might be due to the different rat strains used between studies (diet-induced obese Wistar rats in the present study vs. Zucker Fatty *fa/fa* rats in our previous study [2]). However, when expressed as percentage change in blood glucose levels relative to baseline [16], two-way ANOVA revealed that glycemic control was worse for germ-free mice that received ileal content from RYGB-treated compared with sham-operated rats (*p* = 0.023, main effect of treatment; Figure 1b) in association with a trend toward increased plasma LPS levels (2.56 ± 0.65 EU/mL vs. 1.18 ± 0.19 EU/mL, respectively, *p* = 0.11; Figure 1c). Importantly, body weight was similar between recipient groups (40.4 ± 1.1 g for sham-operated recipients vs. 40.9.56 ± 2.5 g for RYGB-treated recipients, *p* = 0.8; Figure 1d) while epididymal white adipose tissue (eWAT) weight tended to be lower for germ-free mice that received ileal content from RYGB-treated compared with sham-operated rats (3.3 ± 0.38 g vs. 4.4 ± 0.71 g, respectively, *p* = 0.21; Figure 1e). These findings suggest that RYGB-associated ileal microbiota can potentially impair host glycemic control by promoting systemic endotoxemia development. 

### 2.2. Ileal Content of RYGB-Treated Rats Contains Soluble Metabolites Which Disrupt Barrier Function and Structure in Caco-2 Cells

**Figure 2:**
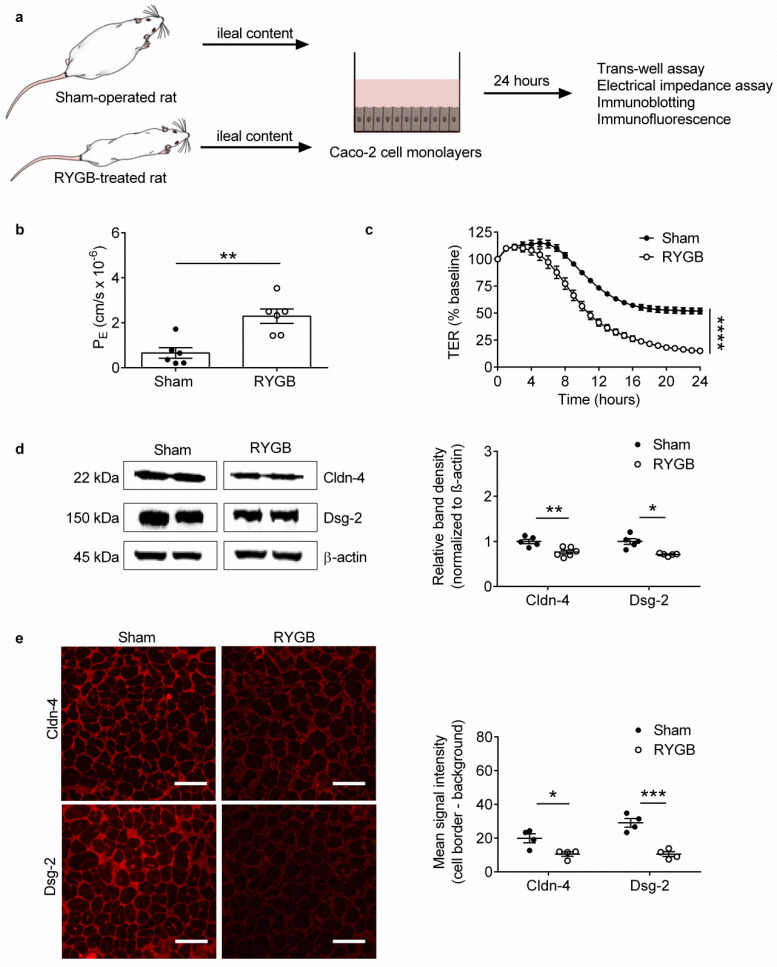
Ileal Content of RYGB-Treated Rats Contains Soluble Metabolites Which Disrupt Barrier Function and Structure in Caco-2 Cells. (**a**) Schematic diagram illustrating experiments performed on Caco-2 cells. (**b**) Permeability coefficients (P_E_) obtained from trans-well assays performed on Caco-2 cells treated for 24 h with ileal content from RYGB-treated and sham-operated rats. *n* = 6 cultures/group from 1 independent experiment. (**c**) Trans-epithelial electrical resistance (TER) values obtained from low-frequency electrical impedance assays performed on Caco-2 cells treated for 24 h with ileal content from RYGB-treated and sham-operated rats. *n* = 10–12 cultures/group from 2 independent experiments. (**d**) Immunoblot analysis of claudin-4 (Cldn-4) and desmoglein-2 (Dsg-2) protein expression in Caco-2 cells treated for 24 h with ileal content from RYGB-treated and sham-operated rats. *n* = 5–6 cultures/group from 1 independent experiment. (**e**) Immunofluorescent analysis of Cldn-4 and Dsg-2 protein levels at the cell border of Caco-2 cells treated for 24 h with ileal content RYGB-treated and sham-operated rats. Scale bar: 25 µm. Data in (**b**–**e**) are presented as mean ± SEM. Statistical significance was determined by two-tailed, unpaired *t*-test with Welch’s correction in (**b**,**d**,**e**) and two-way ANOVA (main effect of treatment) in (**c**). **** *p* < 0.0001, *** *p* < 0.001, ** *p* < 0.01, and * *p* < 0.05.

The IEB serves as a gateway against translocation of pathogenic microbes and toxins from the intestinal lumen to the body [7]. To gain an indication if RYGB-associated ileal microbiota produce and release soluble metabolites which locally disrupt the IEB, we performed functional and structural experiments on Caco-2 cells, a human colonic cancer cell line differentiated into polarized enterocytes, with the same ileal content used on germ-free mice (Figure 2a). 

For our functional experiments, we performed measurements of 4 kDa fluorescein isothiocyanate (FITC)-dextran paracellular flux across Caco-2 cells in trans-well assays, followed by trans-epithelial electrical resistance (TER) measurements between Caco-2 cells in low-frequency electrical impedance assays. This revealed that application of ileal content from RYGB-treated compared with sham-operated rats onto the apical side of confluent Caco-2 cell monolayers for 24 h markedly increased paracellular (apical to basolateral) flux of 4kDa FITC-dextran (P_E_ of 2.29 ± 0.32 cm/s × 10^−6^ vs. P_E_ of 0.65 ± 0.23 cm/s × 10^−6^, respectively, *p* = 0.0021; Figure 2b). Correspondingly, it markedly decreased TER values (1078 ± 106 ohm vs. 3621.9 ± 132 ohm, respectively, *p <* 0.0001; Figure 2c). These results clearly demonstrate that ileal content of RYGB-treated compared with sham-operated rats contains soluble microbiota- and/or host-derived metabolites that have direct, barrier-disruptive effects at the functional level. 

The IEB is comprised of tight junctions, adherens junctions and desmosomes, which are multi-protein complexes localized to the border of enterocytes and establish a tripartite seal between adjacent cells [17,18]. We therefore next performed immunoblot analysis to determine expression of key IEB proteins in Caco-2 cells, followed by high-magnification immunofluorescent analysis to determine their cellular distribution. This revealed that application of ileal content from RYGB-treated compared with sham-operated rats onto confluent Caco-2 cell monolayers decreased expression of the barrier-stabilizing tight junction protein claudin-4 [19] and the barrier-stabilizing desmosomal protein desmoglein-2 [20] (Figure 2d). Similarly, it markedly decreased claudin-4 and desmoglein-2 levels at the cell border (Figure 2e). These results complement the functional findings and demonstrate that ileal content of RYGB-treated compared with sham-operated rats contains soluble microbiota- and/or host-derived metabolites that have direct, barrier-disruptive effects also at the structural level.

## 3. Discussion

The potent and sustained metabolic benefits delivered by RYGB have been partly attributed to favorable shifts in the intestinal microbiota [2,3,5]. We previously found, however, that RYGB-associated ileal microbiota can paradoxically impair host glycemic control [2]. Because of the role of systemic endotoxemia in the etiology of obesity-associated insulin resistance [21], we hypothesized in the present study that microbiota from this part of the gastrointestinal tract produce and release soluble metabolites following RYGB that directly disrupt the IEB. In line with our hypothesis, ileal content from a separate cohort of RYGB-treated compared with sham-operated, diet-induced obese Wistar rats modestly heightened systemic endotoxemia development when transferred to germ-free mice, and compromised barrier integrity when applied onto Caco-2 cells. 

Our findings contrast with those of Wang et al. in which the reduced intestinal permeability in RYGB-treated, diet-induced obese Sprague Dawley rats was attributed to beneficial effects of ileal microbiota-derived metabolites [22]. Specifically, it was proposed that taurine production by ileal microbiota is increased, thereby stabilizing the IEB through upregulation of nucleotide-binding oligomerization domain-like receptor 6 (NLR6) in ileal enterocytes [22]. A possible reason for the discrepancy with our study is that Wang et al. performed co-housing experiments with conventionally raised rats, whereas we performed ileal content transfer experiments with germ-free mice. Additionally, we directly applied ileal content from RYGB-treated and sham-operated rats onto Caco-2 cells to determine effects on barrier function and structure, whereas Wang et al. applied taurine alone (after it was shown to be higher in ileal content of RYGB-treated rats) onto rat ileal explants to determine changes in gene expression of inflammatory mediators [22]. Nevertheless, both experimental approaches incompletely model the post-RYGB ileal microenvironment warranting future studies in which ileal content are applied onto ileal explants/enteroids from the same species. 

We unfortunately could not perform microbiota/metabolomic analysis on our ileal content samples in the present study so the specific RYGB-associated ileal microbiota/metabolites that contribute to disruption of the IEB need to be determined in future studies. Nevertheless, we previously found 6-fold higher levels of *B. Vulgatus* species specifically in ileal content of RYGB-treated compared with sham-operated Zucker Fatty *fa/fa* rats, which have been shown to promote insulin resistance when administered to germ-free mice [23]. Interestingly, a recent metabolomic analysis of content obtained from throughout the gastrointestinal tract of type 2 diabetic female patients revealed that lactose degradation increased specifically in ileal content 3 months after RYGB [24]. If this is similarly the case across species, it could also potentially explain the barrier-disruptive effects of ileal content from RYGB-treated compared with sham-operated rats on Caco-2 cells through increased glucose metabolism [25]. 

An important issue raised by our findings is the failure of ileal content transfer from RYGB-treated rats to consistently impair glycemic control in recipient germ-free mice despite similar protocols and sample sizes across studies [2]. One potential explanation for this inconsistency is that while intestinal permeability may consistently increase in germ-free mice that received ileal content from RYGB-treated rats, only ileal microbiota from the Zucker Fatty *fa/fa* strain, with a formerly more severe dysglycemic phenotype, promotes systemic endotoxemia development. This can be confirmed in future ileal content transfer studies performed on germ-free mice in conjunction with in vivo FITC-dextran and plasma LPS assays. 

As well as favorable shifts in the intestinal microbiota, the improvements in glycemic control caused by RYGB have been partly attributed to an overall attenuation of systemic endotoxemia [11,12,13,14,15]. While the underlying molecular and cellular mechanisms presently remain unclear, we previously found that transfer of jejunal and colonic content from RYGB-treated compared with sham-operated rats to germ-free mice improved oral glucose tolerance in association with suppression of systemic endotoxemia [26]. This suggests that jejunal and colonic microbiota-generated metabolites, including secondary bile acids, overall reduce intestinal permeability following RYGB when glycemic control is improved [26]. In contrast, it can be speculated that RYGB-associated ileal microbiota tip the balance in the other direction during relapse of type 2 diabetes to exert a dominant (negative) effect on systemic endotoxemia, which remains to be formally tested. For those individuals in whom systemic endotoxemia is no longer attenuated postoperatively, approaches that specifically eliminate RYGB-associated ileal microbiota that disrupt the IEB, or block the soluble metabolites that they produce and release, may ultimately provide long-term remission of type 2 diabetes and possibly also confer other metabolic benefits. 

## 4. Materials and Methods

Eleven male Wistar rats (Charles River, Lyon, France) aged 4 weeks were initially group-housed in a dedicated facility with an ambient room temperature of 22 °C and a 12 h light/dark cycle with lights on at 7 a.m. They were immediately placed on a high-fat diet (C1090-60, Altromin, Lage, Germany; 5.2 kcal/g, 60% kcal from fat, 16% kcal from protein, and 24% kcal from carbohydrate) for 5 weeks to induce obesity (body weight of 486.9 ± 11.1 g) and were then randomly allocated to RYGB (*n* = 8; body weight of 488.7 ± 12.9 g) or sham operation (*n* = 6; body weight of 485.1 ± 19.1 g) groups as previously described [27]. Briefly, anesthesia was induced with 5% isoflurane in 2% oxygen and maintained with 2% isoflurane in 2% oxygen. Animals then received 3 mg/kg carprofen analgesia subcutaneously and 1.25 mg/kg amoxicillin antibiotic intraperitoneally. For the RYGB procedure, a midline laporatomy was made and a small gastric pouch 5% of the original stomach size was anastomosed to the lower part of the transected mid-jejunum (15 cm from the pylorus) in an end-to-side fashion to create the alimentary limb. The freed upper jejunum was anastomosed in a side-to-side fashion to the lower jejunum (25 cm from the ileocecal valve) to create the biliopancreatic limb. For the sham procedure, a midline laparotomy was made and a 1 cm longitudinal gastrostomy was performed along the anterior wall of the stomach with subsequent closure. 

Following surgeries, rats were transferred to individual cages and for the first 3 post-operative recovery days were only given a liquid diet (vanilla-flavored Ensure, Abbott Nutrition, Columbus, OH, USA; 0.93 kcal/mL 22% kcal from fat) and 3 mg/kg carprofen analgesia subcutaneously. From post-operative day 4 onwards, rats were given simultaneous free access to both low-fat (Altromin, C1090-10; 3.5 kcal/g, 10% kcal from fat, 24% kcal from protein, and 66% kcal from carbohydrate) and high-fat diets. Energy intake was then measured daily for the next 18 days and averaged for RYGB-treated and sham-operated groups. 

At the 6th post-operative week, rats were euthanized by isoflurane overdose. The gastrointestinal tract was dissected and cut into 10 cm terminal ileal segments. Ileal content was collected in cryotubes, immediately snap-frozen in liquid nitrogen and stored at −80 °C. All procedures were approved by the local regulatory authority (Regierung von Unterfranken: 55.2-2532-2-467).

### 4.1. Ileal Content Transfer Experiments 

Nine germ-free Swiss male mice aged 10 weeks and maintained on an autoclaved, standard chow diet were used for ileal content transfer experiments as previously described [2]. Briefly, the frozen ileal content of RYGB-treated and sham-operated rats were homogenized in phosphate-buffered saline (PBS) supplemented with reducing solution (1% cysteine dissolved in NaHCO_3_ buffer) to preserve anaerobic integrity. The resultant slurries were pooled and orally gavaged (200 µL) into 4 h fasted germ-free mice (*n* = 5 receiving ileal content from RYGB-treated rats and *n* = 4 receiving ileal content from sham-operated rats) who were subsequently maintained in autoclaved individual ventilated cages with sterile bedding and fed autoclaved food and water *ad libitum* for the next 2 weeks. On day 14, an OGTT was performed on 4-h fasted mice by oral administration of 30% d-glucose (2 g/kg body weight). Blood was drawn from the tail vein at −15, 0, 15, 30, 60, 90 and 120 min, and blood glucose levels were measured with a Bayer glucometer. Immediately following the OGTT, plasma was isolated from retro-orbital blood by centrifugation at 5 krpm for 10 min and stored at −80 °C. All procedures were approved by the Danish Animal Research authorities issued by the Danish Committee for Animal Research. Data are expressed as percentage relative to *t* = 0 min for each treatment condition. 

### 4.2. Immunoassays

Plasma LPS was measured using a Limulus Amebocyte Lysate Chromogenic Endpoint Assay (HIT302, HycultBiotech, Uden, The Netherlands) at 1:10 dilution. 

### 4.3. Cell Culture

All cell culture was performed at 37 °C in a 5% CO_2_ and 95% O_2_ atmosphere. Caco-2 cells derived from a colorectal adenocarcinoma of a 72-year old Caucasian male (HTB-37, American Type Cell Culture, Manassas, VA, USA) were grown to confluent monolayers in Dulbecco’s Modified Eagle’s Medium (DMEM) (D5796, Sigma-Aldrich Chemie GmbH, Taufkirchen, Germany) supplemented with 50 U/mL penicillin-G, 50 μg/mL streptomycin, and 10% fetal calf serum (FCS) (Biochrom, Cambridge, UK). For all experiments, FCS was substituted for 10% ileal content. 

### 4.4. Ileal Content Preparation for Cell Culture Experiments

Frozen ileal content was dissolved in distilled water at a concentration of 100 mg/1.5 mL. After vigorous mixing in a TissueLyzer (Qiagen, Hilden, Germany) for 10 min, the resultant slurries were centrifuged at 13 krpm for 10 min at 4 °C. Supernatants were filtered through a 70 µm nylon cell strainer (Falcon Corning, Glendale, AZ, USA), pooled for RYGB-operated and sham-operated groups, and stored in aliquots at −20 °C. 

### 4.5. Measurement of TER

Caco-2 cells were seeded onto 8-well chambers (72040, 8W10E + PET, Applied Biophysics, Troy, NY, USA) and grown to confluent monolayers. DMEM supplemented with ileal content was then applied and low-frequency measurements (400 Hz, 600 s interval), reflecting resistance between cells [28], were immediately made for 24 h using the Electric Cell Impedance Sensor (ECIS) Trans-Filter Adapter 1600R System (Applied Biophysics). Data are expressed as percentage relative to baseline for each treatment condition. 

### 4.6. Measurement of 4 kDa FITC–Dextran Flux 

Caco-2 cells were seeded onto trans-well chambers (353180, 0.4 μm pore size; Falcon) in 6-well plates and grown to confluent monolayers. DMEM supplemented with ileal content was then applied for 24 h followed by incubation for 2 h with fresh clear DMEM (D1145, Sigma) containing 10 mg/mL 4 kDa FITC-dextran (Sigma). Paracellular flux of 4 kDa FITC-dextran was assessed by taking 50 μL aliquots from the outer chamber at baseline and 100 μL aliquots from the inner chamber at 60 min. Fluorescence was measured using a spectrophotometer with excitation and emission wavelengths of 485 nm and 535 nm, respectively. Permeability coefficients (P_E_) were calculated as previously described [29].

### 4.7. Immunoblotting

Caco-2 cells were seeded onto 6-well plates and grown to confluent monolayers. DMEM supplemented with ileal content was then applied for 24 h and Caco-2 cells were homogenized in SDS lysis buffer containing 25 mM HEPES, 2 mM EDTA, 25 mM NaF, 1% SDS and 1% protease inhibitor cocktail (ThermoFisher, Waltham, MA, USA). Protein concentration in cell lysates was determined by the BCA assay (ThermoFisher) and 30 µg protein was resolved by SDS gel electrophoresis and blotted onto nitrocellulose membranes with a pore size of 0.2 µm (ThermoFisher catalogue number LC2009). After blocking in 5% milk, mouse anti-claudin-4 IgG (ThermoFisher catalogue number 32-9400) and mouse anti-desmoglein-2 IgG (ThermoFisher catalogue number 32-6100) primary antibodies were applied onto nitrocellulose membranes at 1/250 dilution in 5% milk/0.1% Tween followed by HRP-labelled goat anti-mouse IgG (catalogue number 115-035-003, Dionova GmbH, Hamburg, Germany) secondary antibody 1/3000 dilution in 5% milk/0.1% Tween. Bound immunoglobulins were visualized by enhanced chemiluminescence (Amersham, London, UK) and quantified by densitometry (ChemicDoc Touch, Bio-Rad Laboratories, Hercules, CA, USA), normalized to β-actin, and expressed relative to the sham-operated group.

### 4.8. Immunofluorescence

Caco-2 cells were seeded onto glass coverslips and grown to confluent monolayers. DMEM supplemented with ileal content was then applied for 24 h onto Caco-2 cells followed by fixing with 2% PFA for 15 min and permeabilisation with 0.1% Triton for 10 min. Mouse anti-claudin-4 IgG (ThermoFisher catalogue number 32-9400) and mouse anti-desmoglein-2 IgG (ThermoFisher catalogue number 32-6100) primary antibodies were then applied at 1/50 dilution followed by Dam Alexa Fluor 488-conjugated (ThermoFisher catalogue number A-21201) secondary antibody at 1/200 dilution before mounting on glass microscope slides with Vectashield mounting medium (catalogue number VEC-H-1500, Biozol Diagnostica Vertreib GmbH, Eching, Germany). Fluorescence images with a field of view (FOV) of 135 µm × 135 µm were generated using a LSM 780 confocal microscope (Zeiss, Oberkochen, Germany) at 60× magnification. Image analysis was performed on four random FOVs for each treatment condition with Image J software (https://imagej.nih.gov/ij/index.html (accessed on 15 December 2019)). Signal intensity was measured along 8µm lines drawn orthogonally across cell borders and averaged. Background signal intensity was similarly measured in the intracellular space, averaged, and subtracted from the average signal intensity across cell borders. The resulting curves were then integrated to obtain mean signal intensities for each FOV. 

### 4.9. Statistics

Data are presented as mean ± S.E.M. All statistical tests were performed on Prism 8.0 (Graphpad Software, San Diego, CA, USA). Two-tailed, unpaired *t*-test and two-way ANOVA were applied where indicated. A *p* value of ≤0.05 was considered statistically significant. 

## Data Availability

The data in this study are available in the article.

## Supplementary Materials

The following are available online at https://www.mdpi.com/2218-1989/11/3/153/s1, Figure S1: Effect of Ileal Content Transfer from RYGB-Treated Rats on Oral Glucose Tolerance in Recipient Germ-Free Mice.
