# Supplementary material for: Leaky Gut as a Potential Culprit for the Paradoxical Dysglycemic Response to Gastric Bypass-Associated Ileal Microbiota
Source: Metabolites. 2021 Mar 8;11(3):153. doi: 10.3390/metabo11030153 (PMC7998592; doi:10.3390/metabo11030153)
Supplement: Supplementary file 1 [file metabolites-11-00153-s001.pdf]

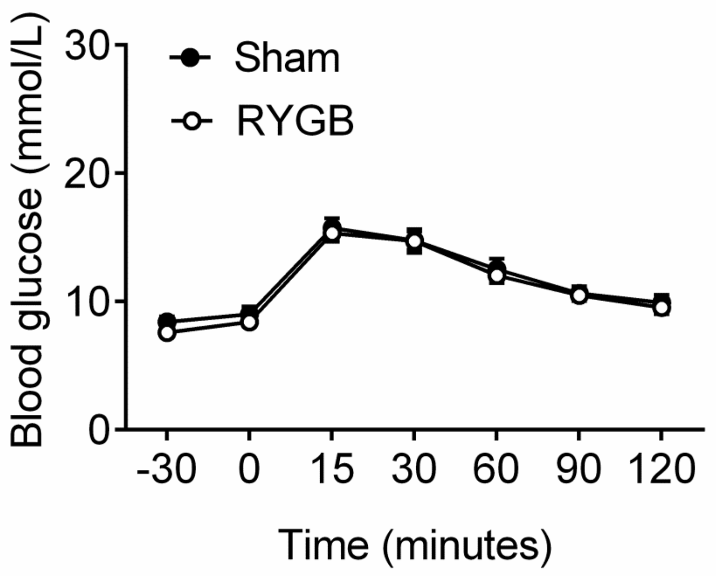

Figure S1. Effect of Ileal Content Transfer from RYGB-Treated Rats on Oral Glucose Tolerance in Recipient Germ-Free Mice Tail vein blood glucose concentrations during an oral glucose tolerance test in germ-free mice that had received ileal content from RYGB-treated and sham-operated rats. n = 4–5 mice/group. Data are presented as mean ± SEM.
